# A Rare De Novo *RAI1* Gene Mutation Affecting BDNF-Enhancer-Driven Transcription Activity Associated with Autism and Atypical Smith-Magenis Syndrome Presentation

**DOI:** 10.3390/biology7020031

**Published:** 2018-05-24

**Authors:** Clemer Abad, Melissa M. Cook, Lei Cao, Julie R. Jones, Nalini R. Rao, Lynn Dukes-Rimsky, Rini Pauly, Cindy Skinner, Yunsheng Wang, Feng Luo, Roger E. Stevenson, Katherina Walz, Anand K. Srivastava

**Affiliations:** 1 John P. Hussman Institute for Human Genomics, University of Miami, FL 33136, USA; cabad@med.miami.edu (C.A.); LCao@med.miami.edu (L.C.); n.rao@miami.edu (N.R.R.);; 2 J. C. Self Research Institute of Human Genetics, Greenwood Genetic Center, Greenwood, SC 29646, USA; mcook@ggc.org (M.M.C.); ldrimsky@ggc.org (L.D.-R.); rpauly@ggc.org (R.P.); cds11251977@gmail.com (C.S.); res@ggc.org (R.E.S.); 3 Molecular Diagnostic Laboratory, Greenwood Genetic Center, Greenwood, SC 29646, USA; juliejones@ggc.org; 4 School of Computing, Clemson University, Clemson, SC 29634, USA; yunshew@g.clemson.edu (Y.W.); luofeng@clemson.edu (F.L.); 5 Dr. John T. Macdonald Foundation Department of Human Genetics, Miller School of Medicine, University of Miami, FL 33136, USA; 6 Department of Genetics and Biochemsitry, Clemson University, Clemson, SC 29634, USA

**Keywords:** autism spectrum disorder, Smith-Magenis syndrome, *RAI1*, neurodevelopmental disorder, mutation

## Abstract

Deletions and mutations involving the Retinoic Acid Induced 1 (*RAI1*) gene at 17p11.2 cause Smith-Magenis syndrome (SMS). Here we report a patient with autism as the main clinical presentation, with some SMS-like features and a rare de novo *RAI1* gene mutation, c.3440G > A (p.R1147Q). We functionally characterized the RAI1 p.R1147Q mutant protein. The mutation, located near the nuclear localization signal, had no effect on the subcellular localization of the mutant protein. However, similar to previously reported RAI1 missense mutations in SMS patients, the RAI1 p.R1147Q mutant protein showed a significant deficiency in activating in vivo transcription of a reporter gene driven by a BDNF (brain-derived neurotrophic factor) intronic enhancer. In addition, expression of other genes associated with neurobehavioral abnormalities and/or neurodevelopmental disorders were found to be altered in this patient. These results suggest a likely contribution of RAI1, either alone or in combination of other factors, to social behavior and reinforce the *RAI1* gene as a candidate gene in patients with autistic manifestations or social behavioral abnormalities.

## 1. Introduction

Smith-Magenis syndrome (SMS, MIM 182290) is a rare multiple congenital anomaly and developmental disability syndrome with an estimated incidence of 1:15,000–1:25,000 live births [1]. The syndrome is commonly characterized by a specific combination of clinical features, including distinctive craniofacial appearances, skeletal anomalies, speech and motor delay, a variable degree of intellectual disability, sleep disturbances, and self-injurious and/or aggressive attention-seeking behavior [1,2].

Autism spectrum disorder (ASD) is characterized by significant deficits in reciprocal social interactions, impaired communication and restricted, repetitive behaviors. ASD commonly co-occurs with other developmental, psychiatric, psychological, and neurological abnormalities. Patients with SMS have also been described to display stereotypies, sensory integration difficulties and social responsiveness scale scores consistent with ASD [3,4,5,6]. Typically, a deletion of chromosome 17p11.2, which encompasses multiple genes, including the retinoic acid-induced 1, *RAI1*, gene or a heterozygous mutation in the *RAI1* gene, cause SMS [7,8,9,10,11,12,13,14,15,16]. Mice carrying an inactivated *Rai1* allele (*Rai1*^+/−^) showed most of the SMS features and also showed diminished interest in social odors, abnormal submissive tendencies, and increased repetitive behaviors suggesting a role of *Rai1* in social behavior in mice [17,18,19]. However, findings of aberrant social behaviors in mice carrying deletions or duplications of multiple genes, including the *Rai1* gene, need to be interpreted with caution [20].

RAI1 is a transcription factor whose transcript has been found in multiple regions of the human brain including neurons, but not glia, of the dentate gyrus and cornus ammonis of the hippocampus [21]. Recently, it has been shown that murine Rai1 preferentially occupies DNA regions near active promoters and enhances the expression of genes involved in cell-cell neuronal communication [22]. A critical role for Rai1 in normal neural and craniofacial development has also been shown in *Xenopus* [23]. Morpholino-mediated *Rai1* knockdown resulted in defects in the developing brain and face in *Xenopus* and such brain defects were also correlated with a decrease in the neurotrophic factor, bdnf [23]. Interestingly, *Bdnf* was also found to be downregulated in Rai1 heterozygous mice [24]. In addition, several Rai1-target genes were found to be involved in cell adhesion, axon guidance and neuronal morphogenesis [22] and Rai1 was shown to bind *Bdnf* and *Htr2c* [22]. We have shown that the human wild type RAI1 protein has nuclear localization and transcription factor activity [25,26,27]. Moreover, several mutant RAI1 proteins showed significant differences in the transcription factor activity compared with the wild type [27].

Here we describe a patient with autism, and some additional clinical features commonly present in patients with SMS including hyperactivity, obsessive-compulsive-like actions, aggressiveness, irregular eating and abnormal sleep patterns, who carries a de novo missense mutation, c.3440G > A (p.R1147Q), in the *RAI1* gene. Functional studies of the p.R1147Q mutant protein revealed a significant impairment in recognizing and activating transcription of a reporter gene driven by a BDNF enhancer, described as an endogenous target sequence for RAI1 [24], when compared to the wild type form. In addition, by RNA-seq, we show that other genes associated with neurodevelopmental/neurobehavioral abnormalities are apparently altered in this patient or have abnormal expression levels, suggesting that his atypical SMS-clinical presentation might be the result of a complex genetic makeup.

## 2. Materials and Methods

### 2.1. Patient Report

**Figure 1:**
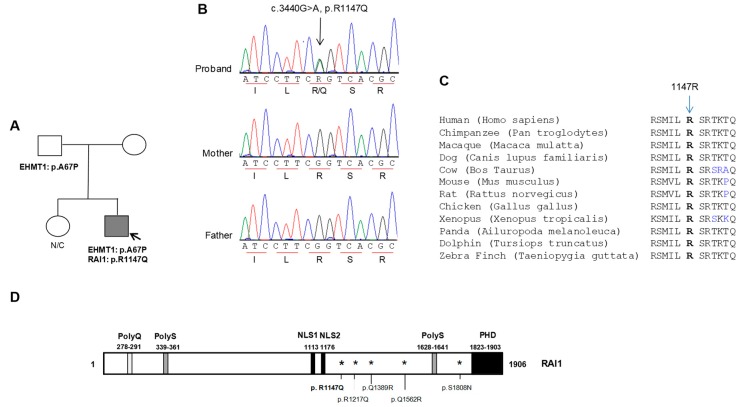
Molecular analyses of a patient with autism. (**A**) A partial pedigree of a family with one child with autism. Nonsynonymous variants identified in the proband and his parents are shown. Grey filled symbol, affected autism; (**B**) Automated sequence chromatograms showing the *RAI1* gene variation (arrow) in the proband. Triplet codon (underlined) and translated amino acids are shown; (**C**) An alignment of a region of human RAI1 showing the highly conserved 1147R residues (in bold type) altered in the proband; (**D**) Schematic representation of the domains (PolyQ, PolyS, nuclear localization signals (NLS1 and NLS2) and the PHD domain) contained in the RAI1 protein. The p.R1147Q alteration identified in this study is shown in bold. In addition, the previously described and functionally tested p.R1217Q, p.Q1389R, p.Q1562R and p.S1808N mutations are depicted.

A partial pedigree of the Caucasian family with one child with autism is shown in Figure 1A. Both parents are of normal intelligence and behavior. The proband is a 20-year-old male who began walking at 9 months of age and started using words at the usual time or a little late. At 16 months of age, he had an episode when he was inconsolable. Subsequent to that time, over the next three months, there was a noticeable decline. He stopped using words altogether and had essentially no social interaction. He began roaming around and was in constant motion, and seemed more self-absorbed. He was diagnosed as having ASD using CARS (Childhood Autism Rating Scale) and met criteria for ASD as outlined in DSM-IV [28,29]. He was toilet trained at 5 years of age. He had a febrile seizure at 5 years of age and subsequently has experienced nonfebrile tonic-clonic seizures which sleep deprivation seemed to precipitate. He had no EEG but his cranial CT and cranial MRI at 5 years of age were normal. His craniofacial features were normal. However, he appeared to have wide-set eyes. He had three café-au-lait spots and several pigmented nevi and a hypopigmented macule on his right arm. He had no abnormalities or deformities of the upper and lower limbs. At 9 years of age, the proband’s father noted that his son appeared to be different from other children with autism and described him as having more of a movement disorder with boundless energy, hyperactivity to the point of exhaustion and obsessive-compulsive-like actions. Although not temperamentally aggressive, he was found to be aggressive at times and had very irregular eating and sleeping patterns.

### 2.2. Mutation Screening

Next Generation Sequencing (NGS) of 62 autosomal and X-linked genes (Table S1) known for their association with ASD and/or syndromes that include ASD as a significant clinical feature was performed. Five micrograms of the patient’s genomic DNA sample was fragmented to 4–6 kb using a Covaris S220 Focused-Ultrasonicator (Covaris, Inc., Woburn, MA, USA). RainDance enrichment for the 62 genes was performed on the RDT1000 instrument (RainDance Technologies, Lexington, MA). A standard fragment library for analysis by SOLiD sequencing was prepared for the sample. Library amplification was performed using emulsion PCR, and the products of the emulsion PCR were purified (AMPure) and then deposited onto a glass slide for analysis by the 5500xl system (Applied Biosystems, Foster City, CA, USA). After bioinformatically processing the data, a tertiary analysis was performed using NextGENe software (SoftGenetics, State College, PA, USA). All changes deemed of potential clinical relevance were confirmed by Sanger sequencing.

### 2.3. Plasmid Constructs

The full-length clone of human *RAI1-HA* cDNA (accession numbers for human *RAI1*: NM_030665 and Q7Z5J4) was previously constructed [26]. The mutant *RAI1-HA* c.3440G > A was generated with site-directed mutagenesis by overlapping PCR utilizing the primers Forward 1 5′-GCAAAGAAAGAGCCTGTGCC-3′ and Reverse 1 5′-GTTTTGGTGCGTGACTGAAG-3′ for PCR 1 and Forward 2 5′-CTTCAGTCACGCACCAAAAC-3′ and Reverse 2 5′ CTCCTCTTCTTAGGCGCCAG-3′ for PCR 2. Following this, a third PCR was made using PCR 1 and PCR 2 as templates with Forward 1 and Reverse 2 primers. The mutated fragment was subcloned to replace the wild type sequence in the full length *RAI1* cDNA with the enzymes *AfeI* and *PfiMI*. All clones were verified by DNA sequencing for the presence of the desired mutation and no extra change in the nucleotide sequence. For expression analysis, the cDNA of the mutant form was subcloned into the pALTER-MAX vector (Promega Corporation, Madison, WI, Madison, WI, USA). The constructs were sequenced in order to confirm the mutations and the absence of extra nucleotide changes.

### 2.4. Cell Culture

Neuro-2a cells were grown in Dulbecco’s Modified Eagle Medium supplemented with 10% fetal bovine serum, penicillin (100 units/mL), streptomycin (100 µg/mL) at 37 °C with 5% CO_2_ until 95% confluence was attained.

### 2.5. Western Blot and Immunofluorescence Analysis

To study the expression of the mutant protein, Neuro-2a cells were transfected with Lipofectamine 2000 (Invitrogen, Carlsbad, CA, USA) and the plasmid pALTER-MAX *RAI1-HA* wild type or *RAI1-HA* c.3440G > A. All transfections were performed according to the manufacturer’s protocol. Western blot analysis was performed as previously described [26]. Immunodetection was performed using rat anti-HA (1:7000, Roche, Branford, CT, USA) and rabbit anti-β-tubulin (1:1000, sc-9104 Santa Cruz, Dallas, TX, USA). Results were visualized by chemiluminescence. For immunofluorescence, cells were fixed 24 h after transfection with 4% paraformaldehyde followed by permeabilization with 0.2% Triton X-100 in PBS. Subcellular localization of RAI1-HA wild type and mutant forms were detected using the anti-HA high affinity antibody (1:1000, clone 3F10, Roche, Branford, CT, USA). Secondary antibody conjugated to Alexa Fluor 488 (1:1000) was used. Cells were stained with 4′, 6-diamidino-2-phenylindole, dihydrochloride (DAPI) and mounted with a Dako fluorescent mounting medium.

### 2.6. Reporter Gene Assays

In order to evaluate the transactivation activity of RAI1, transient transfections in Neuro-2a cells were performed in 35 mm plates with Lipofectamine 3000 (Gibco, Life Technologies, Carlsbad, CA, USA). The amounts of plasmid DNA used were defined according to manufacturer’s protocol. GAL4-BD fusions of human *RAI1* wild type and mutant form were co-transfected with the luciferase reporter plasmid pFR-Luc (Agilent Technologies, La Jolla, CA, USA). For normalizing the results in case of transfection efficiency variations, the vector pSV-β-galactosidase (Promega Corporation, Madison, WI, USA) was also co-transfected for expression of β-galactosidase. After 24 h post-transfection, the cells were lysed and the luciferase activity was measured with Luciferase Assay Kit (Agilent Technologies, La Jolla, CA, USA) according to manufacturer’s instructions. The Relative Light Units (RLUs) were measured in duplicate in a luminometer (Turner BioSystems 20/20n, Promega Corporation, Madison, WI, USA). The β-galactosidase activity of the extracts was measured using the microassay protocol of β-galactosidase Assay kit (Agilent Technologies, La Jolla, CA, USA). Each assay was carried out in duplicate. For assessing the ability of RAI1 and its mutant form for activating transcription through the *BDNF* enhancer, the plasmid pGL3-*BDNF* enhancer previously generated was utilized [27] plus the pSV-β-Galactosidase (Promega Corporation, Madison, WI, USA) and pAlter-MAX *RAI1-HA* wild type or mutant form were co-transfected in Neuro-2a cells. At 24 h post-transfection, cells were lysed for measuring, in duplicate, luciferase and β-galactosidase activities as described previously [27]. Cells co-transfected with pGL3 *BDNF* enhancer, pSV-β-Galactosidase and empty pAlter-MAX vector were used to normalize the data to basal *BDNF* activation levels.

### 2.7. Statistical Analysis

Statistical analysis was performed utilizing the two-tailed Student’s *t*-test, and a *p*-value ≤0.05 was considered significant.

### 2.8. Next Generation RiboZero RNA Sequencing and RNA-Seq Data Analysis

Total RNA was extracted from lymphoblastoid cell lines from the proband and 9 age-, sex- and race-matched normal control individuals using mirVana™ miRNA Isolation Kit (LifeTech, part number AM1560, Carlsbad, CA, USA). Yield (absorbance at 260 nm) and purity (260/280 = 1.8–2.0, 260/230 ≥ 1.7) were assessed with a NanoDrop^®^ (Thermo Scientific, Waltham, MA, USA) spectrophotometer. Automated capillary electrophoresis with the RNA 6000 Nano kit on an Agilent BioAnalyzer 2100 was used to measure RNA integrity. All samples had an RNA integrity number (RIN) of 8 or above. Sequencing was performed at the Medical University of South Carolina Genomic Center (Charleston, SC, USA). 100–200 ng of total RNA was used to prepare RNA-Seq libraries using the TruSeq^®^ Stranded Total RNA RiboZero Sample Prep Kit (Illumina, San Diego, CA, USA) following the protocol as described by the manufacturer. Paired-end cluster generation was performed on the cBot as described by the manufacturer (Illumina, San Diego, CA, USA). Clustered RNA-seq libraries were paired-end sequenced with 2 × 125 cycles on an Illumina HiSeq2500 using v4 chemistry. Demultiplexing from base calls was performed utilizing Illumina’s bcl2fastq software to generate FASTQ files. The sequenced reads for each sample were obtained as FASTQ files. After performing sample QC on raw FASTQ files, determining any necessary trimming that may be required using FASTQC and applying some internally developed scripts, reads were aligned to Human Reference Genome (hg19) using HISAT2 that uses the Hierarchical Graph FM index (HGFM). VARSCAN2, and ANNOVAR were used for variant calling and annotation respectively. A false positive-filter was applied to all variants that included examination of the number of variant reads, base quality, position in read of variant, and strand bias. Variants were also filtered by AAF ≤ 0.05 in gnomAD, 1000G, NHLBI and dbSNP; additional filters were applied from SIFT, PolyPhen, MutationTaster, Mutation Assessor and CADD. Finally, we applied a phenotype filter using HPO terms to further reduce the gene list. Gene counts were imported into R (version 3.3.2) using featureCounts [30]) from the Rsubread package. Normalization and differential expression analysis was performed based on the difference in gene counts between the proband and controls using DESeq2. DESeq2 estimates differential expression using a negative binomial model which has been shown to reduce false positives compared to other methods. The DESeq2 model also internally corrects for library size. The BH FDR (false discovery rate) was calculated to control for multiple hypothesis testing where the log2 fold change and Wald test adjusted *p*-value were also calculated for each gene. Gene set enrichment analysis was performed to identify gene ontology terms associated with altered gene expression for the comparisons performed. We filtered the results by *padj* ≤ 0.05, sorted by log2FoldChange and highlighted the top genes.

## 3. Results

We characterized a 20-year-old male patient (Figure 1A) who was diagnosed as having autism at 2 years of age and met the DSM-IV criteria [28]. High-resolution chromosome analysis revealed a normal 46, XY karyotype and chromosome morphology appeared normal by G-banding. No deletions or duplications of known or of potential significance were detected by microarray analysis using the Affymetrix CytoScan HD microarray.

In addition to autism, the proband had other clinical features, such as cognitive impairment, motor delay, sleep disturbance, aggressive behavior, hyperactivity, and irregular eating patterns, as commonly seen in patients with the *RAI1* gene deletions or mutations (Table 1). Thus, we performed next generation diagnostic panel sequencing that included the *RAI1* gene and an additional 61 autosomal and X-linked genes (Table S1) known for their association with ASD and/or syndromes that include autism as a significant clinical feature. We identified two heterozygous non-synonymous alterations: a c.199G > C alteration in the Kleefstra syndrome gene (*EHMT1*) [31] and a c.3440G > A alteration in the *RAI1* gene (Figure 1A,B). The alteration in the *EHMT1* gene is predicted to cause an amino acid change from an alanine to a proline at position 67 (p.A67P). The *RAI1* gene alteration is predicted to result in an amino acid change from an arginine to a glutamine at position 1147 (p.R1147Q). The alterations were not reported in the Human Gene Mutation Database or the public SNP databases including the 1000 Genome Project variation data, dbSNP, NHLBI Exome Variant Server and the Ensembl variation database. Both variants were listed in ExAC data set (allele frequency: 0.00001659; 2/120,544 and no homozygotes for the *EHMT1* variant; allele frequency: 0.00001665; 2/120,118 and no homozygotes for the *RAI1* variant), a compilation of unrelated individuals sequenced as part of various disease-specific and population genetic studies. Parental DNAs were analyzed by targeted Sanger sequencing. The *EHMT1* gene alteration (c.199G > C, p.A67P) was inherited from the unaffected healthy father (data not shown and Figure 1A) and the proband’s unaffected healthy mother carried the wildtype *EHMT1* allele (c.199G/G), suggesting a likely benign nature for this variant in the proband. The *RAI1* gene variant was not identified in the healthy parents (Figure 1A,B), indicating that it had occurred de novo. The residue involved in the RAI1 p.R1147Q alteration was found to be highly conserved (Figure 1C) and located near one of the two nuclear localization signals (NLS) (Figure 1D). Bioinformatic analyses using PolyPhen2, SIFT, and CADD indicated the p.R1147Q variant to be pathogenic or not tolerated.

A comparison of phenotypes between the patient described in this study and patients harboring the common 17p11.2 deletion and those with intragenic mutations within *RAI1* is shown. The percentages of occurrence for each of the phenotypes are given. +, phenotype present; -, absent or not reported; &, normal development then sudden decline; #, irregular eating patterns. The percentages in the table are a summary of 77 patients harboring the common deletion and 23 of patients carrying a mutation in *RAI1* [3,5,9,10,16,26].

### 3.1. Functional Characterization of the RAI1-p.R1147Q Mutant Protein

**Figure 2:**
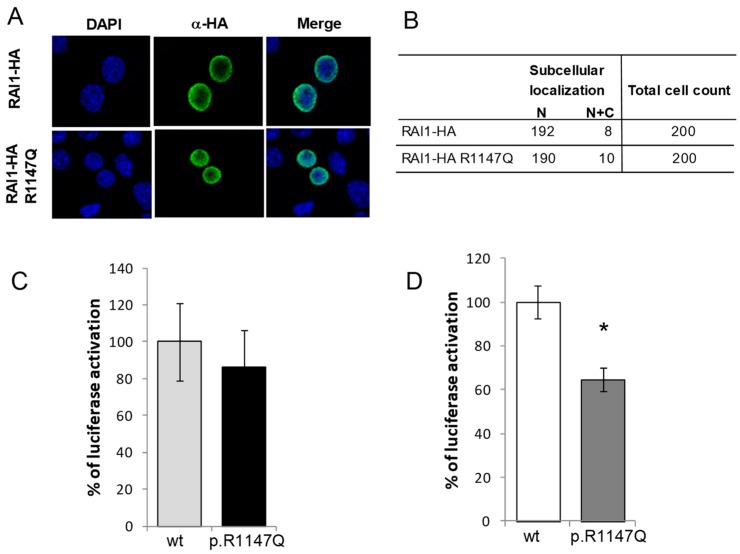
In vitro evaluation of RAI1-p.R1147Q mutant. (**A**) Plasmid coding for the wild type or the mutant form of RAI1 was transfected in Neuro-2a cells and immunofluorescence was performed with anti-HA (green) antibody, while nuclei staining is shown with DAPI; (**B**) The subcellular localization (nuclear or cytoplasmic) of 200 positive cells for the immunodetection is summarized in the table; (**C**) The percentage of the reporter transcription activation is shown for RAI1-HA wild type (light grey column, N = 4) and p.R1147Q mutant (black column, N = 4); (**D**) Neuro-2a cells were co-transfected with a BDNF fused luciferase reporter plasmid, a β-galactosidase reporter plasmid, and either RAI1 wild type (white column, N = 3) or RAI1 p.R1147Q (dark grey column, N = 3). Twenty four hours post-transfection, the reporter proteins were measured from the cell lysates; (**C**,**D**) Activation of the reporter for the RAI1 wild type was considered 100% for normalization. Values represent mean ± SEM. (* = *p* < 0.05).

To examine the pathogenic potential for the RAI1 c.3440G > A variant, we characterized the hemagglutinin epitope (HA)-tagged RAI1 wild type and RAI1-p.R1147Q mutant proteins. RAI1-HA and RAI1-HA c.3440G > A clones were transiently transfected in Neuro 2a cells as described in Materials and Methods. To address whether there was a difference in stability or final molecular weight of the mutated protein, a Western blot analysis of the cell lysates was performed. In both cases, we detected a protein band of ~250 kDa, similar to the wild type RAI1 (data not shown). The subcellular localization of RAI1 p.R1147Q was evaluated by immunofluorescence against the HA epitope, in Neuro-2a cells transfected with pAlter-MAX *RAI1-HA* or *RAI1*–*HA* c.3440G > A, respectively. No difference in the subcellular localization of the mutant RAI1 protein was observed. Both the wild type and mutant proteins were primarily localized to the nucleus (Figure 2A,B).

We next evaluated the transcription factor activity with co-transfection of a plasmid coding for a fusion protein GAL4 DNA binding domain-RAI1 (wild type or the mutated form) and a plasmid containing the GAL4-binding element upstream of the luciferase coding sequence [15,26,27]. RAI1-HA p.R1147Q showed an increment of 86 ± 20% of activation of luciferase activity compared to the wild type protein, which was considered to be 100% activation (Figure 2C). This change was not significant (*p* = 0.6), indicating that the mutated proteins retained the comparable transcription factor capacity. Together, these results indicated that the p.R1147Q variant apparently had no effect on the synthesis, subcellular localization, or transcription factor activity of the RAI1 protein.

Next, we tested the capacity of the mutant form in recognizing and activating the transcription of a reporter gene driven by a *BDNF* intronic enhancer sequence previously recognized as an endogenous target sequence for RAI1 [24]. This intronic sequence was cloned into the pGL3-promoter vector, which contains an SV40 promoter upstream of the luciferase coding sequence (pGL3 BDNF enhancer or enhancer/reporter plasmid). Twenty-four hours after transfection of Neuro-2a cells with the enhancer/reporter plasmid and an empty vector pAlter-Max (as control), there was an activation of 26-fold over the basal expression level of the reporter luciferase, due to the presence of endogenous RAI1 (data not shown). When the enhancer/reporter plasmid was transfected together with the construct pAlter-MAX RAI1-HA, the transcription of the luciferase gene increased ~860 fold. However, when the enhancer/reporter plasmid was co-transfected with the construct carrying the missense mutation p.R1147Q, the transcription of the luciferase gene was induced ~250 fold over the basal activity, significantly less than the wild type RAI1 protein *p* = 0.01 (Figure 2D), indicating a deficiency of the mutant protein to recognize the BDNF enhancer sequence.

### 3.2. Additional Variations and Altered Expression of Genes Related with Neurobehavioral Outcomes

In addition to autism, the clinical presentation of the proband was in part consistent with only some features of SMS. To understand whether additional genes were implicated in the final clinical presentation, we checked for other gene alterations and evaluated for abnormal expression of genes that might contribute to the final phenotype by RNA-seq comparing the proband vs. age-, sex- and race-matched normal individuals. The *RAI1* mutation was confirmed by the RNA-seq data (Table S2A), indicating that the mutated allele is expressed in the proband. In addition, several other heterozygous variants of unknown significance were found in transcripts of genes related to neurobehavioral and/or neurodevelopmental phenotypes (Table S2B), adding to the growing evidence that multiple genetic changes might influence the final clinical presentation to some degree. Several of these variants (e.g., in *TTI2*, *ASPM*) were rare and bioinformatically found to be deleterious or pathogenic in nature. However, mutations in these genes were previously reported to be recessive and thus it is unlikely that any of these heterozygous variants are of clinical significance in the proband (Tables S2A and S2B) [32,33].

Table 2 lists the top differentially expressed genes between the proband sample and controls (see also Table S3). The RNA-seq analysis included only 1 patient compared to 9 matched control samples, and thus the finding of differential expressed genes needs to be undertaken with caution and requires further verification in additional similar cases. Nonetheless, among downregulated genes (adjusted *p* value < 0.002), interestingly enough, DST, a gene previously related with autism was found to be altered in this patient and also was found to be dysregulated in a mouse model for SMS syndrome [17]. Several additional genes also appeared to be significantly altered in their expression in the proband (Table 2 and Table S3). Functional analysis using GOrilla (http://cbl-gorilla.cs.technion.ac.il/) and DAVID [34] showed that one of the top dysregulated genes, *DST*, was enriched for the category of cellular component Gene Ontology term, GO:0031673 (H zone), which is a part of the contractile fiber of the A band. DST is a cytoskeletal linker protein that integrates intermediate filaments such as actin and microtubule cytoskeleton networks. Defects in this gene are associated with sensory and/or autonomic abnormalities. *PTCH1*, an important Hedgehog (Hh) signaling pathway receptor (OMIM *601309) gene, is also shown to be differentially expressed and was enriched with a biological process GO term GO:0010157 (response to chlorate). The Hh pathway plays an important role in embryonic development and adult stem cell functioning and thereby it remains a possibility that aberrant expression of some of these genes either directly or indirectly might influence the phenotypic expression in the proband.

## 4. Discussion

Smith-Magenis syndrome is a neurodevelopmental disorder with a clinical presentation including craniofacial dysmorphic features, abnormalities of sleep-wake circadian rhythm, and cognitive impairment with behavioral symptoms [1]. In addition, individuals with SMS have also been described to have stereotypies, sensory integration difficulties and social communication problems consistent with select features of ASD [4,6]. Several heterozygous *RAI1* gene mutations (nonsense, missense and frameshift mutations) have been found to be associated with SMS [7,8,9,10,11,12,13,14,15,16]. The *RAI1* gene has also been indirectly associated with spinocerebellar ataxia (SCA2), schizophrenia, and autism [35,36,37]. Recently, a mutation in the *RAI1* gene has been identified in a morbidly obese child diagnosed with ROHHAD Syndrome [38]. When neuropsychiatric behaviors are associated with a CNV, it can be difficult to ascribe them to a single gene within the CNV [20]. However, *RAI1* has already been validated by several groups and studies, in humans and mice, to be responsible for the main characteristics in SMS, including some of the neurobehavioral aspects of the syndrome. Furthermore, mice carrying an inactivated *Rai1* allele (*Rai1*^+/−^) showed an increase in stereotypic behaviors and abnormal social interactions when compared with wild type littermates [18], further suggesting that *Rai1* is related with social behavior. We have identified a rare de novo RAI1 missense mutation, p.R1147Q, in a patient diagnosed with autism by DSM-IV. Additional clinical features present in the proband and overlapping with features present in SMS individuals (Table 1) included hyperactivity, obsessive-compulsive-like actions, aggression and irregular eating and sleep patterns. SMS can be associated with deletions in 17p11.2 or mutations in the *RAI1* gene. Comparison of the clinical presentation of our patient with those carrying a common deletion in 17p11.2 or mutations in the *RAI1* gene (Table 1) suggests that proband does not present with classical SMS, but is rather an atypical SMS clinical presentation. Of note, this patient carries an extra change in the *EHMT1* gene that seems not to be pathogenic by in silico prediction analysis, and is shared with his father, who has not been reported to have an abnormal phenotype. *EHMT1* mosaicism in apparently unaffected parents was recently found to be associated with autism spectrum disorder and neurocognitive dysfunction [39]. In our case, the mutation in *EHMT1* is not mosaic in the proband or his father. However, we cannot discount that it might be adding to the final clinical phenotype in the patient. The p.R1147Q mutation was not reported in the Human Gene Mutation Database or the public SNP databases, including the 1000 Genome Project variation data, but was listed at a very low minor allele frequency (0.00001665; 2/12,0118 and no homozygotes) in the ExAC dataset. Bioinformatic analyses predicted the mutation to be pathogenic. Furthermore, the p.R1147 residue was found to be highly conserved and located near one of the two nuclear localization signals (NLS). Two main functional domains for the RAI1 protein have been defined: the N-terminal half with the transactivational activity, and the C-terminal half responsible for the nuclear localization of the protein [26]. In vitro analysis of the p.R1147Q mutant protein in Neuro-2a cells showed no altered subcellular localization or transactivational activity. Bdnf, a neurotrophic growth factor, whose reduced expression has been identified in depression, schizophrenia and obsessive-compulsive disorder in human and animal models [40], was also found to be downregulated in *Rai1*^+/−^ mice [24]. A *Bdnf* intronic enhancer was found to be an endogenous target sequence for Rai1. We have shown that the p.R1147Q mutant protein demonstrated a significant deficiency in recognizing and activating the transcription of a reporter gene driven by the BDNF intronic enhancer. Previously identified RAI1, and functionally tested missense mutations located within the C-terminal half of RAI1—p.R1217Q, p.Q1389R, p.Q1562R and p.S1808N (Figure 1D)—produced similar effects, as they were able to induce transcription of the reporter gene driven by the *BDNF* intronic enhancer but significantly less than the wild-type RAI1 protein [26,27]. This suggests that all of these mutations are affecting RAI1 direct or indirect binding to DNA or interfering with the transcription factor activity regulation of the protein. Interestingly, murine Rai1 binds to the promoter region of *Bdnf* [22].

Transcript identification and quantification of differential gene expression by RNA-seq for clinical use has recently been proven to be a powerful tool [41]. RNA-seq of this patient sample allowed us not only to confirm that the pathogenic *RAI1* allele was expressed but also to recognize that other genes, such as *DST*, *TTI2*, and *ASPM* that are related with neurobehavioral abnormalities, were differentially expressed or altered in the proband [14,32,33,42]. Five case-specific loss-of-function variants were identified in the *DST* gene following the sequencing of 215 synaptic genes in 147 cases with ASD, 273 cases with schizophrenia, and 287 controls [42]. Mutations in the *TTI2* gene have been associated with autosomal recessive ID and the *ASPM* gene has been implicated in autosomal recessive primary microcephaly-5 [32,33]. The finding of a functionally abnormal mutation on *RAI1* in a patient with ASD further supports the notion of its role in social behavioral abnormalities complementing previous findings in mice [18]. Interestingly enough, in the DECIPHER database (https://decipher.sanger.ac.uk/), we found that one of nine cases with the *RAI1* SNV (1bp insertion) had autistic features in the described clinical presentation. Studies in additional subjects would be required to examine any such association. Taken together, our findings suggest that the *RAI1* gene mutations may contribute to some cases of autism. In addition, the present work supports the concept of a complex genetic makeup in patients with diverse clinical presentation.

## 5. Conclusions

Our data shows that the *RAI1* gene mutation is also responsible for atypical Smith-Magenis syndrome presentation and could be a contributing factor to the social abnormalities, and reinforces the notion that *RAI1* should be considered as a candidate gene in children with autistic manifestations or social abnormalities.

## Figures and Tables

**Table 1:** Comparison of the clinical presentation in the proband compared to SMS patients with common deletion and *RAI1* mutations.

	SMS Patients		
Phenotypes	% In Common 17p11.2 Deletion [3,5,10,16,26]	% In *RAI1* Mutations [3,9,10,16,26]	PROBAND p.R1147Q
**Craniofacial Abnormalities**	100	100	-
**Skeletal Abnormalities**			
Short stature	70–80	11	-
Scoliosis/vertebral abnormalities	73	40–50	-
Short broad hands/brachydactyly	85	88	-
**Otorhinolaryngolocial**			
Hoarse voice	66–80	76–86	-
Hearing loss	60–68	11–33	-
**Neurological**			
Cognitive impairment	100	100	+
Infantile hypotonia	<90	50–61	-
Speech delay	>90	70	+ ^&^
Motor delay	>90	60–70	+ ^&^
Sleep disturbance	90	100	+
EEG abnormalities	50–66	80	Not examined
Seizures	11–30	16.6–50	+
**Behavioral**			
Self-hugging	50–80	100	-
Onychotillomania	25–85	80–100	-
Polyembolokoilamania	25–85	75–80	-
Head banging/face slapping	70	90	-
Hand biting	80	60–71	-
Attention seeking	80–100	100	+
Aggressive behavior		55	+
Self-injurious behavior	70–90	>90	-
Hyperactivity	80	100	+
Autistic features	90^(6)^	NR	+(DSM-IV)
**Other features**			
Cardiac defects	30	0	-
Renal/urinary tract defect	30	0	-
Obesity	18	78	-
Overeating	25	81	+ ^#^

**Table 2:** Genes with altered expression in the proband.

Gene	log2FoldChange	Description	*p* Value	*padj*
*GNG12*	1.719707224	Upregulated in the Patient sample	4.81 × 10^−9^	5.02 × 10^−5^
*HCP5*	1.617432091	Upregulated in the Patient sample	3.37 × 10^−7^	0.001757018
*GGACT*	1.35253954	Upregulated in the Patient sample	1.74 × 10^−8^	0.000120784
*HAPLN3*	1.269895566	Upregulated in the Patient sample	2.34 × 10^−5^	0.044419951
*ICOS*	1.228236573	Upregulated in the Patient sample	2.58 × 10^−9^	5.02 × 10^−5^
*TRIM26*	0.991872296	Upregulated in the Patient sample	1.60 × 10^−8^	0.0371298
*PKHD1L1*	−0.998015573	Downregulated in the patient sample	8.08 × 10^−7^	0.002466581
*DST*	−1.109285101	Downregulated in the patient sample	5.29 × 10^−7^	0.002209546
*CCZ1*	−1.313033217	Downregulated in the patient sample	2.33 × 10^−5^	0.044419951
*MTRNR2L8*	−1.319578464	Downregulated in the patient sample	2.83 × 10^−6^	0.007389326
*PTCH1*	−1.559860559	Downregulated in the patient sample	8.27 × 10^−7^	0.002466581

## Supplementary Materials

The following are available online at http://www.mdpi.com/2079-7737/7/2/31/s1, Table S1: Genes included in the syndromic autism panel, Table S2A: Variants of unknown significance identified in transcripts of genes in the proband, Table S2B: Genes with variant of unknown significance and related to neurobehavioral and/or neurodevelopmental phenotypes, Table S3: Genes with altered expression in the proband.
