# Supplementary material for: A Rare De Novo RAI1 Gene Mutation Affecting BDNF-Enhancer-Driven Transcription Activity Associated with Autism and Atypical Smith-Magenis Syndrome Presentation
Source: Biology (Basel). 2018 May 24;7(2):31. doi: 10.3390/biology7020031 (PMC6023015; doi:10.3390/biology7020031)
Supplement: Supplementary file 1 [file biology-07-00031-s001.zip › Table S1-S3 05182018/Table S1 05112018.docx]

Table S1: Genes included in the syndromic autism panel.

| AP1S2 | CNTNAP2 | FOXP1 | MEF2C | PAFAH1B1 | RELN | UBE3A |
| --- | --- | --- | --- | --- | --- | --- |
| ARX | COH1 | FOXP2 | MET | PCDH19 | SCN1A | ZEB2 |
| ATRX | CREBBP | GABRB3 | MID1 | PHF6 | SHANK3 |  |
| AVPR1A | DHCR7 | HOXA1 | NHS | PNKP | SLC2A1 |  |
| BDNF | DMD | HPRT1 | NIPBL | PQBP1 | SLC6A4 |  |
| BRAF | EHMT1 | KDM5C | NLGN3 | PTCHD1 | SLC9A6 |  |
| CACNA1C | FGD1 | L1CAM | NLGN4X | PTEN | SMC1A |  |
| CASK | FMR1 | MBD5 | NRXN1 | PTPN11 | TCF4 |  |
| CDKL5 | FOLR1 | MECP2 | NSD1 | RAB39B | TSC1 |  |
| CHD7 | FOXG1 | MED12 | OPHN1 | RAI1 | TSC2 |  |
